# FROM ANXIETY TO NEURONAL RESCUE: EXPLORING THE POTENTIAL OF LORAZEPAM IN TRAUMATIC BRAIN INJURY

**DOI:** 10.51894/001c.122920

**Published:** 2024-08-31

**Authors:** Luke Noah

**Affiliations:** 1 College of Osteopathic Medicine Michigan State University

## 48

### INTRODUCTION/BACKGROUND

Traumatic brain injury (TBI) is a highly devastating event that places a significant burden on the worldwide healthcare system. In the US, TBI accounts for around 2.5 million emergency department visits, nearly 300,000 hospitalizations, and over 50,000 deaths annually (Flanagan, 2015). Despite this high burden, effective neuroprotective therapies following TBI are lacking with little in the drug development pipeline. A newly described critical part of TBI pathology is Ferroptosis, a regulated cell death pathway that results from accumulation of lipid oxidation products (Kenny et al., 2019). The loss of GPR68 exacerbates both stroke severity in animal models and causes ferroptosis in Glioblastoma cells, establishing a neuroprotective GPR68-Ferroptosis axis (Wang et al, 2020; Williams et al. 2023). The FDA-approved benzodiazepine Lorazepam has been indicated to selectively bind to and activate GPR68 as a positive allosteric modulator (Cornwell et al. 2023).

### OBJECTIVES/HYPOTHESIS

We hypothesize that agonists of GPR68, including Lorazepam, can protect neurons from cell death following TBI by mitigating ferroptotic death. This neuroprotective action of GPR68 can translate to improved outcomes from neurological trauma and potentially improve survival and quality of life.

### METHODS

TBI models -1. In vitro- Oxygen Glucose Deprivation (OGD), Glutamate Excitotoxicity; 2. In vivo- Larval Zebrafish Brain Injury. Markers of neuronal cell death: Lactate dehydrogenase release and cell viability assays. Markers of ferroptosis: Lipid peroxidation and TFRC expression.

## RESULTS

Our lab has identified a highly specific small molecule inhibitor of GPR68, ogremorphin (OGM) (in press), that promotes ferroptosis in an ATF4 dependent manner. These pro-ferroptosis effects are attenuated by siRNA-mediated knockdown of GPR68. Moreover, preliminary data from our lab suggests that activation of GPR68 can attenuate ferroptosis in cells undergoing ferroptosis.

### DISCUSSION/CONCLUSIONS

Impairments seen in TBI are largely thought to be mediated by neuronal cell death through cell-mediated ferroptosis. Pharmacological activation of GPR68, attenuating ferroptosis could be used in the treatment of TBI or other types of neuronal injury. Through our findings, we hope to put forward a mechanism for effective and efficient TBI treatment.

